# Supplementary material for: Arabic within culture forensic interviews: Arabic native speaking lay-observer truth and lie accuracy, confidence, and verbal cue selection
Source: PLoS One. 2024 Sep 23;19(9):e0310384. doi: 10.1371/journal.pone.0310384 (PMC11419357; doi:10.1371/journal.pone.0310384)
Supplement: S1 Appendix — (DOCX) [file pone.0310384.s001.docx]

**Appendix A: Interview Protocol**

**Phase 1 Explain and Rapport**

The interviewer explained to the participant that she/he was aware that a laptop had been seriously damaged and so would be asking a series of questions about what had occurred. The interviewer provided a general overview of the interview process and explained four ground rules (report everything; do not guess; say if you do not understand; say if you do not know the answer to a question).

Participants were then offered the chance to ask questions. The interviewer then engaged the interviewee in informal conversation to build rapport. Rapport building conversation continued for a minimum of 2 mins at this point during which the interviewer led the participant to understand the reciprocal nature of the interview, using silences and active listening to encourage the participant to speak/respond following self-disclosure statements made by the interviewer.

**Phase 2** **Free Account**

The second phase commenced with the interviewer providing a positively-framed moral rationale [73]. The following prompts were delivered slowly, with a 5 second pause between each prompt: *“I am going to ask you to tell me in more detail what happened earlier. Telling me about everyone that was involved and everything that happened when the laptop was damaged will* ***make*** *you feel that you are doing something to ensure that innocent people do not end up getting blamed for the damage”.*

*“I have found that even those people who did not initially want to tell me everything understand that the best way forward is when everyone exercises their responsibility to provide full details”.*

*“Those who have told me everything have done just that – exercised their responsibility, and that is the right and proper thing to do – they have done the right thing”*

*“Telling me everything is what you ought to do, too. That way I can fully understand what happened. Would you agree”?*

*“So, if we are in agreement, please have good think about what happened and answer all my questions in as much detail as you can. Thank you”*

*“What I would like you to do now is to tell me exactly what happened from the time you entered the room until the time you left, in as much detail as possible. Remember the four ground rules I described earlier - report everything; do not guess; say if you do not understand; say if you do not know the answer to a question”*

The free account was uninterrupted by the interviewer. Once the interviewee had finished speaking the interviewer silently waited a further 5 seconds (counting silently to 5) before thanking the interviewee and moving into the questioning phase of the interview. During this account the interviewer displayed attentive listening behaviours.

**Phase 3 Probed Questions**

This phase comprised four information-gathering segments concerning *people, actions*, *verbal* and *mistakes*.

1. People involved *“Describe to me absolutely everything about everyone involved in the damage to the laptop. Please try and provide as much detail as possible because I was not there, and so I don’t know what happened”.*
2. Movements. *“Can you talk me through everyone’s movements in as much detail as possible. Please try and provide as much detail as possible because I was not there, and so I don’t know what happened”*
3. Verbal *“Describe everything that was said by everyone in the room. It doesn’t matter if you can’t remember everything, but try hard to explain who said what, and who spoke to whom. Thank you”*
4. Mistakes. *‘Just before we finish, I wonder, have you left anything out or made any mistakes in what you have told me about what happened in the classroom? Please take the time to think hard about what happened and tell me everything. It is really*

*important that I understand.*

**Phase 4 Challenge.** Irrespective of information yield and/or interview performance, interviewees were verbally challenged concerning the completeness of the account given thus far, and ‘pushed’ for more information:

*‘I think I have a fair understanding of what has happened, but I am not sure that you have told me everything you know. I have interviewed others who were in the room at the same time and they have provided me with more information than you have. It is important that you tell me as much as you can because otherwise I cannot fully understand what has happened. Take a few minutes and have another think about what happened. Tell me everything’.*

The interviewer sat silently waiting for the interviewee to respond for up to 10 seconds (counting silently). If the interviewee responded, then the interviewer sat and listened. No extra questions were asked. If the interviewee did not respond after 10 seconds then the interviewer moved seamlessly to the next phase.

**Phase 5 Closure** This final phase marked the end of the interview. Here the interviewer explained that the interview had now finished and thanked the participant for taking part. The participant was offered the opportunity to ask any questions. The recording device was turned off.

**Appendix B: Materials**

**No-Cue Direction Instructions**

Next you will be asked to click on a link, to access a video of an interview, conducted in Arabic.

The interview lasts about 10 mins and concerns a serious incident that took place on a UK University campus. The interviewer is trying to find out what happened and who was responsible. 

During a research data collection session, a university laptop computer was seriously damaged.

**It is known that the person being interviewed was in the room where the incident occurred and saw what happened.**
During the interview the interviewee answers questions and explains what happened. Please take your time to watch and listen carefully because you will then be asked to answer some questions about the interview in two ways.

First, evaluate the answers given by the interviewee when questioned about the serious incident on **detailedness** using a scale from 0 to 10.

1 = (no detailed information was provided) 5 = (some detailed event information was provide) 10 = (a lot of detailed information about the event)

Detailedness is defined as:

“The degree to which the interviewee includes details such as descriptions of people, places, actions, objects, events, and the timing of events; the degree to which the message of the interviewee seemed complete, concrete, striking, or rich in details”.

Link to film

Detailedness judgment scale here (0 to 10)

Now you have made a *detailedness* judgement, decide whether the interviewee is lying or telling the truth using the following rule: If you judged detailedness to be 6 or more, you should determine the interviewee is telling the truth. OR if you judged detailedness it to be 5 or lower, you should determine the interviewee is lying about what happened. Truths are typically more detailed than lies. Based on your detailedness judgement, please indicate whether the interviewee is a truthteller (detailedness of 6 or more) or liar (detailedness of 5 or less).

Liar or Truthteller (forced choice answer)

**No Cue Direction Instructions**

Next you will be asked to click on a link, to access a video of an interview, conducted in Arabic.

The interview lasts about 10 mins and concerns a serious incident that took place on a UK University campus. The interviewer is trying to find out what happened and who was responsible. 

During a research data collection session, a university laptop computer was seriously damaged.

**It is known that the person being interviewed was in the room where the incident occurred and saw what happened.**
During the interview the interviewee answers questions and explains what happened. Please take your time to watch and listen carefully because you will then be asked to answer some questions about the interview.

Link to film

Liar or Truthteller (forced choice answer)

**Appendix C: Observer Cultural/Ethnicity Self Identity Data**

| **Cultural Self Identity** | **Number (percentage)** |
| --- | --- |
| Israeli Arab | 27 (12.40) |
| European Arab | 11 (5.11) |
| African/Black African/North African | 63 (29.03) |
| Arab | 42 (19.36) |
| Middle Eastern | 47 (21.66) |
| Nigerian | 12 (5.52) |
| West Asian | 15 (6.92) |
