# Supplementary material for: Arabic within culture forensic interviews: Arabic native speaking lay-observer truth and lie accuracy, confidence, and verbal cue selection
Source: PLoS One. 2024 Sep 23;19(9):e0310384. doi: 10.1371/journal.pone.0310384 (PMC11419357; doi:10.1371/journal.pone.0310384)

PLOS ONE

Arabic within culture investigative interviews: Arabic native speaking lay-observer truth and lie accuracy, confidence, and verbal cue selection.

--Manuscript Draft--

**Manuscript Number:**	PONE-D-24-12342
**Article Type:**	Research Article
**Full Title:**	Arabic within culture investigative interviews: Arabic native speaking lay-observer truth and lie accuracy, confidence, and verbal cue selection.
**Short Title:**	Arabic within culture native speaking truth and lie accuracy
**Corresponding Author:**	Coral Dando, Ph.D.<br>University of Westminster<br>London, UNITED KINGDOM
**Keywords:**	Arabic native speaker; within culture veracity; investigative interviews: truth and lie accuracy; Confidence; verbal cue selection
**Abstract:**	Cultural differences in behavioral and verbal norms and expectations can undermine credibility in cross cultural interactions, often triggering a lie bias. However, research in this domain is heavily North American and Western European centric, and so how individuals from non-western cultures infer veracity, the cues they attend to, and the decisions they make is not well understood. We report novel research investigating native Arabic speakers' truth and lie judgments having observed a matched native language investigative interview with a mock person of interest. 217 observers viewed a truthful or a deceptive video interview and were either directed to attend to detailedness of the interviewee's responses as a cue to veracity or given no cue direction. Irrespective of condition, a truth bias (66% accuracy) emerged, but observers were most accurate (79%) in the truth condition. However, accuracy dropped to just 23% when instructed to use the detailedness cue when judging deception. The truthful interviewee was rated as more plausible and more believable than the deceptive interviewee. Verbal veracity cues attended too were constant across conditions with 'corrections' emerging as the most important veracity cue. Our results are mixed in terms of deviating from the findings of others in some areas, while offering some constant findings. Despite limitations, this research raises a challenge for research and practice in forensic interview contexts worldwide whereby western centric psychological understanding may not be robust for non-western within cultural interviews.
**Order of Authors:**	Coral Dando, Ph.D.
	Alexandra Sandham, PhD
	Charlotte Sibbons, MSc
	Paul J. Taylor, PhD
**Additional Information:**	
**Question**	**Response**
**Financial Disclosure**<br><br>Enter a financial disclosure statement that describes the sources of funding for the work included in this submission. Review the submission guidelines for detailed requirements. View published research articles from *PLOS ONE* for specific examples.	Yes

Powered by Editorial Manager® and ProduXion Manager® from Aries Systems Corporation

This statement is required for submission and **will appear in the published article** if the submission is accepted. Please make sure it is accurate.<br><br>**Funded studies**<br>Enter a statement with the following details:<br>• Initials of the authors who received each award<br>• Grant numbers awarded to each author<br>• The full name of each funder<br>• URL of each funder website<br>• Did the sponsors or funders play any role in the study design, data collection and analysis, decision to publish, or preparation of the manuscript?<br><br>Did you receive funding for this work?	
Please add funding details.<br>as follow-up to "**Financial Disclosure**<br><br>Enter a financial disclosure statement that describes the sources of funding for the work included in this submission. Review the submission guidelines for detailed requirements. View published research articles from *PLOS ONE* for specific examples.<br><br>This statement is required for submission and **will appear in the published article** if the submission is accepted. Please make sure it is accurate.<br><br>**Funded studies**<br>Enter a statement with the following details:<br>• Initials of the authors who received each award<br>• Grant numbers awarded to each author<br>• The full name of each funder<br>• URL of each funder website<br>• Did the sponsors or funders play any role in the study design, data collection and analysis, decision to publish, or preparation of the manuscript?<br><br>Did you receive funding for this work?"	This work was funded by the U.S. Government's High-Value Detainee Interrogation Group (HIG) awarded to the first author - Contract DJF-16-1200-V-0000737. Statements of fact, opinion and analysis in the article are those of the authors and do not reflect the official policy or position of the HIG or the U.S. Government.

Powered by Editorial Manager® and ProduXion Manager® from Aries Systems Corporation

Please select the country of your main research funder (please select carefully as in some cases this is used in fee calculation).<br>as follow-up to "**Financial Disclosure**<br><br>Enter a financial disclosure statement that describes the sources of funding for the work included in this submission. Review the submission guidelines for detailed requirements. View published research articles from *PLOS ONE* for specific examples.<br><br>This statement is required for submission and **will appear in the published article** if the submission is accepted. Please make sure it is accurate.<br><br>**Funded studies**<br>Enter a statement with the following details:<br>• Initials of the authors who received each award<br>• Grant numbers awarded to each author<br>• The full name of each funder<br>• URL of each funder website<br>• Did the sponsors or funders play any role in the study design, data collection and analysis, decision to publish, or preparation of the manuscript?<br><br>Did you receive funding for this work?"	UNITED STATES - US
**Competing Interests**<br><br>Use the instructions below to enter a competing interest statement for this submission. On behalf of all authors, disclose any competing interests that could be perceived to bias this work—acknowledging all financial support and any other relevant financial or non-financial competing interests.<br><br>This statement is required for submission and **will appear in the published article** if the submission is accepted. Please make sure it is accurate and that any funding	The authors have declared that no competing interests exist

sources listed in your Funding Information later in the submission form are also declared in your Financial Disclosure statement.<br><br>View published research articles from *PLOS ONE* for specific examples.<br><br>**NO authors have competing interests**<br><br>Enter: *The authors have declared that no competing interests exist.*<br><br>**Authors with competing interests**<br><br>Enter competing interest details beginning with this statement:<br><br>*I have read the journal's policy and the authors of this manuscript have the following competing interests: [insert competing interests here]*<br><br>* typeset	
**Ethics Statement**<br><br>Enter an ethics statement for this submission. This statement is required if the study involved:<br><br>• Human participants<br>• Human specimens or tissue<br>• Vertebrate animals or cephalopods<br>• Vertebrate embryos or tissues<br>• Field research<br><br>Write "N/A" if the submission does not require an ethics statement.<br><br>General guidance is provided below. Consult the submission guidelines for detailed instructions. **Make sure that all information entered here is included in the Methods section of the manuscript.**	This research was ethically approved by the University of Westminster Research Ethics Committee: ETH1617-0528 and the US Dept of Justice FBI institutional Review Board Docket No. 353-16 and was conducted in accordance with the British Psychological Society and Health and Care Practitioner Council codes of ethical conduct.

**Format for specific study types**<br><br>**Human Subject Research (involving human participants and/or tissue)**<br>• Give the name of the institutional review board or ethics committee that approved the study<br>• Include the approval number and/or a statement indicating approval of this research<br>• Indicate the form of consent obtained (written/oral) or the reason that consent was not obtained (e.g. the data were analyzed anonymously)<br><br>**Animal Research (involving vertebrate animals, embryos or tissues)**<br>• Provide the name of the Institutional Animal Care and Use Committee (IACUC) or other relevant ethics board that reviewed the study protocol, and indicate whether they approved this research or granted a formal waiver of ethical approval<br>• Include an approval number if one was obtained<br>• If the study involved *non-human primates*, add *additional details* about animal welfare and steps taken to ameliorate suffering<br>• If anesthesia, euthanasia, or any kind of animal sacrifice is part of the study, include briefly which substances and/or methods were applied<br><br>**Field Research**<br><br>Include the following details if this study involves the collection of plant, animal, or other materials from a natural setting:<br>• Field permit number<br>• Name of the institution or relevant body that granted permission	
**Data Availability**<br><br>Authors are required to make all data underlying the findings described fully available, without restriction, and from the time of publication. PLOS allows rare exceptions to address legal and ethical concerns. See the PLOS Data Policy and FAQ for detailed information.	No - some restrictions will apply

Powered by Editorial Manager® and ProduXion Manager® from Aries Systems Corporation

A Data Availability Statement describing where the data can be found is required at submission. Your answers to this question constitute the Data Availability Statement and **will be published in the article**, if accepted.<br><br>**Important:** Stating 'data available on request from the author' is not sufficient. If your data are only available upon request, select 'No' for the first question and explain your exceptional situation in the text box.<br><br>Do the authors confirm that all data underlying the findings described in their manuscript are fully available without restriction?	
**Describe where the data may be found in full sentences. If you are copying our sample text, replace any instances of XXX with the appropriate details.**<br><br>• If the data are **held or will be held in a public repository**, include URLs, accession numbers or DOIs. If this information will only be available after acceptance, indicate this by ticking the box below. For example: *All XXX files are available from the XXX database (accession number(s) XXX, XXX.*).<br>• If the data are all contained **within the manuscript and/or Supporting Information files**, enter the following: *All relevant data are within the manuscript and its Supporting Information files.*<br>• If neither of these applies but you are able to provide **details of access elsewhere**, with or without limitations, please do so. For example:<br><br>*Data cannot be shared publicly because of [XXX]. Data are available from the XXX Institutional Data Access / Ethics Committee (contact via XXX) for researchers who meet the criteria for access to confidential data.*<br><br>*The data underlying the results presented in the study are available from (include the name of the third party*	All raw data files (SPSS data sheets) will be available from the OSF database.

and contact information or URL).<br>• This text is appropriate if the data are owned by a third party and authors do not have permission to share the data.<br><br>* typeset	
Additional data availability information:	Tick here if the URLs/accession numbers/DOIs will be available only after acceptance of the manuscript for publication so that we can ensure their inclusion before publication.

Powered by Editorial Manager® and ProduXion Manager® from Aries Systems Corporation

Manuscript

Click here to access/download;Manuscript;Arabic Observor Study Final Submited PLOSONE April 16 2024.doc

1. I am sorry that I cannot correct the statistical method, I only correct or review the rationality of the research. I don't master this method of statistical research

Arabic within culture investigative interviews: Arabic native speaking lay-observer truth and lie accuracy, confidence, and verbal cue selection.

Coral J. Dando[1]

Alexandra L. Sandham [2]

Charlotte Sibbons [1]

Paul J. Taylor [3]

[1], University of Westminster, Dept of Psychology, London, UK

[2] The Open University, Milton Keynes. UK

[3] Lancaster University, Dept. of Psychology, Lancaster, UK

Address for correspondence:

Correspondence concerning this article should be addressed to Coral J. Dando, School of Psychology, University of Westminster, 115 New Cavendish Street, London W1W 6UW, United Kingdom. E-mail: c.dando@westminster.ac.uk

Key Words: Arabic; Investigative Interview, Deception, Verbal Cues, Lay-Observer

Abstract

Cross cultural differences in behavioral and verbal norms and expectations can undermine credibility, often triggering a lie bias. However, research in this domain is heavily North American and Western European centric, and so how individuals from non-western cultures infer veracity, the cues they attend to, and the decisions they make is not well understood. We report novel research investigating native Arabic speakers' truth and lie judgments having observed a matched native language investigative interview with a mock person of interest. 217 observers viewed a truthful or a deceptive video interview and were either directed to attend to detailedness of the interviewee's responses as a cue to veracity or given no cue direction. Irrespective of condition, a truth bias (66% accuracy) emerged, but observers were most accurate (79%) in the truth condition. However, accuracy dropped to just 23% when instructed to use the detailedness cue when judging deception. The truthful interviewee was rated as more plausible and more believable than the deceptive interviewee. Verbal veracity cues attended too were constant across conditions with 'corrections' emerging as the most important veracity cue. Our results are mixed in terms of deviating from the findings of others in some areas, while offering some constant findings. Despite limitations, this research raises a challenge for research and practice in forensic interview contexts worldwide whereby western centric psychological understanding may not be robust for non-western within cultural interviews.

2. add research methods in the abstract.

Acknowledgements

This work was funded by the U.S. Government's High-Value Detainee Interrogation Group (HIG) awarded to the first author - Contract DJF-16-1200-V-0000737. Statements of fact, opinion and analysis in the article are those of the authors and do not reflect the official policy or position of the HIG or the U.S. Government.

We would like to thank Amelia Dickinson for her work as a part-time Research Assistant, data manager, and coder on this project.

Introduction

Persons of interest is a term used by many law enforcement organisations when referring to individuals suspected of knowing investigation relevant information (IRI), but who at that point in time may not be suspected of criminal activity themselves [1, 2]. For example, a person of interest may have witnessed an incident, have some information about a crime that has occurred or is being planned, or might have a close relationship with a person suspected of criminal wrongdoing. Persons of interest are questioned by professionals worldwide using various interview techniques to yield IRI [3, 4, 5]. Although not required to make decisions regarding the veracity of accounts provided *per se*, interviewers are expected to be alert to deception. Accordingly, they seek to maximize opportunities to detect truth and lies, gathering information, challenging and/or accepting responses to questions as appropriate, seeking clarification, and deciding when to terminate an interview and when to persist [6, 7, 8].

Following an interview with a person of interest, various parties not involved in the interview process itself become concerned with understanding the integrity of accounts provided. Examples include professional non-investigators (e.g., legal and criminal justice employees) and lay-persons (e.g., jury members, assessment panels comprising non-investigative professionals), who observe interviews or portions of interviews [3, 9, 10]. Understanding lay-persons judgement behaviours is vital since they can be involved in decisions centered on whether to further investigate, the likelihood of a successful prosecution, judging guilt or innocence, and whether responses to cross examination during court proceedings correspond with accounts provided in the interview, for example.

Lay-persons have no investigative experience nor expertise, they simply observe and listen. Nonetheless, lay-observers are an important element of many criminal and civil

investigations and formal proceedings worldwide [11, 12, 13]. The psychological deception literature concerning lay-observer judgements of truth and lies is generally consistent. Lay-observers have a propensity to attend to pseudo diagnostic verbal and non-verbal veracity cues and consequently often exhibit a truth bias [14, 15, 16, 17] or at best perform at around chance levels (50: 50) when judging veracity [18, 19, 20, 21, 22]. That said, psychological understanding of lay observer judgments is primarily centered on north American and western European English-speaking interactions [23, 24, 25]. How lay observers from non-western cultures infer veracity, the cues they attend to, and the decisions they make is not well understood. Yet, culturally defined truth and lie cues do exist [25, 26, 27, 28] and so it is sensible to investigate non-western lay-observer behaviours since individuals from different cultures may behave variously.

Lay-persons truth and lie judgments are the focus of the research reported here, but rather than focusing on cross-cultural interactions, we investigate truth and lie judgments in matched culture, matched native language contexts, where interviews are conducted in Modern Standard Arabic (henceforth, Arabic). Twenty-eight nations in western Asia have Arabic as their official language with an estimated 420 million native Arabic speakers globally, yet we found minimal relevant research, and as far as we can ascertain the veracity judgement behaviours of lay observers from this significant cultural group have been neglected.

3. Do the authors summarize the main research question and key findings?
4. Do the authors identify other literature on the topic and explain how the study relates to this previously published research?

Deception and Culture

Cultural differences can be defined as ‘the collective programming’ that distinguishes one group from another [29]. Hence, culture typically refers to customary beliefs, values and characteristic societal markers that determine individual attitudes, and shape individual and social behaviours [30, 31, 32]. While individuals from different cultures do behave and communicate variously [27, 28, 33, 34] all deceivers face a similar challenge, that is how to

appear credible. In investigative interview contexts, deceivers may embed lies in the truth and so offer a partially truthful account that includes some deceptive elements, and/or they withhold chunks of information completely, often suggesting a lack of recall or denying having seen or heard elements of the events in question [3, 35, 36]. Cross cultural understanding of similarities and differences in truth and lie behaviours in interview contexts continues to emerge. However, irrespective of culture, lying is often more cognitively demanding than telling the truth [3, 37, 38, 39, 40, 41] and so the cognitive challenges of formulating and maintaining a deceptive account in response to investigative questioning appear robust and pancultural.

To appear credible, all deceivers must first formulate a sensible lie and maintain their lie script in response to questions, while holding back the truth to avoid slips of the tongue. Liars must also respond appropriately to follow-up clarification or challenge questions as an interview progresses. Clarification can be in the form of asking for further event details or asking interviewees to repeat answers to monitor consistency. Challenges in an interview typically concern directly questioning whether a response is veridical, or the interviewer might introduce information that has not previously been revealed which casts doubt on the veracity of previous responses. Either way, deceptive interviewees must manage several enduring concurrent cognitive processes [41, 42, 43, 44] throughout an interview. In contrast, telling the truth requires the interviewee to recall and verbalise an experienced event, which invokes fewer cognitive resources.

Despite widespread perceptions to the contrary, physical behaviours such as gaze avoidance, self-adaptors, and nervousness have not been found to be reliable cues to veracity because of individual, contextual and cultural variances (Dando et al., 2022; Sandham et al., 2022; Vrij & Vrij 2020). However, several observable verbal behaviours predicated by cognitive load theories of deception and the impact of attempting to manage several

concurrent cognitive processes to appear credible have begun to emerge as potentially stable cues, irrespective of culture and/or language. Deceiver verbal accounts are often impoverished and simple, whereby they provide fewer event specific details than truth-tellers (Dando et al., 2022: 2023; Ormerod & Dando, 2015; Sandham et al., 2022; Vrij & Vrij 2020). Although *appearing* to provide informative accounts, in fact deceivers often offer general knowledge information rather than detailed event details that could be verified, for example. Guiding observers to rate detailedness and the amount of verifiable event information has been found to support improved detection of deception performance beyond chance for observers in some circumstances (Levine et al., 2007).

Truthful accounts are also often more plausible, that is they sound more sensible, likely, or believable. Judgements of plausibility, in terms of how 'believable' an account is, have been found to distinguish truth tellers from liars in some contexts whereby plausibility ratings for deceptive accounts are typically lower than for truthful accounts [25, 26, 35, 45, 46]. Furthermore, observer plausibility ratings have been reported to positively predict the detailedness of an account [35, 36]. Lay-observers may, therefore, be able to recognise variable verbal behaviours such as detailedness and plausibility for guiding them in making real-time truth and lie judgments. However, questions arise centred on the real-world challenges for lay-observers.

Cut-off indicators do not exist for the amount of detail an interviewee may or may not provide in response to individual questions or across the interview as a whole, and so subjective assessments are necessary [35, 36]. Likewise, plausibility and believability also require subjective assessments concerning how individuals understand how sensible or likely an account is [46]. Subjective assessments are guided by cultural norms, expectations and understandings and so it is important to investigate whether these emerging cues to truth and lies are intersubjective [47]. That is, whether context germane to culture leverages space that

might make detailedness and plausibility less important or less valid subjective cues for some cultures.

Second, applied researchers use various paradigms whereby lay-observer participants often view multiple interviews or read multiple interview transcripts where some participants have been incentivised to be deceptive while others are instructed to be truthful [26, 35, 38, 42, 48, 51]. This approach has been vital for improved understanding of the efficacy of various types of interview approaches for amplifying cues to truth and lies, and how veracity judgments are made. However, in reality lay observers may only have access to a single interview (audio, transcript or video), or one or two relevant portions of an interview which does not allow group performance comparisons. Access to just one interview does not support relative nor comparative verbal behaviour judgements as often occurs in laboratory research paradigms where anomalous verbal behaviour can sometimes be easier to spot across a group, or cohort [49, 50]. In such instances, observers can be alerted to truth and lies in a manner that does not map onto the real world where they may be asked to make an absolute judgment based on the behaviours apparent in one interview or transcript.

The Current Research

Using first language as a proxy for cultural origins, the research reported here seeks to advance understanding of the utility of detailedness, believability and plausibility as veracity cues in forensic interview contexts for lay-observers from a non-western culture. To reflect real-world conditions, observers have access to just one video interview conducted in Arabic, following which they are immediately asked to make a real-time truth or lie judgment. Despite Arabic being one of the most spoken languages worldwide, as far as we can ascertain, lay-observer truth and lie judgments of this significant cultural group have received little attention using single culture paradigms. Here, interviewees are mock persons of interest, known to have IRI. Interviews are conducted in Arabic using an information

gathering interview protocol. Lay-observers all have Arabic as their first language and are asked to self-identify their ethnic/cultural background.

The directly relevant psychological literature is sparce and research findings are mixed, but cultural differences in perceptions of deception are reported [23, 27, 28, 52, 53, 54] and verbal behaviours vary across cultures. These cultural differences in behavioural norms and expectations can undermine credibility in cross-cultural interview contexts, whereby misunderstanding and misinterpretation typically triggers a lie bias, although not always [55, 56]. The cognitive challenges associated with verbal deception are believed to be pancultural, and so verbal behaviours triggered by cognitive demand, such as levels of event detailedness, believability and plausibility, for example, may transcend culture and language. While individuals from Western individualist cultures tend to provide more information than those from non-Western collectivist cultures [57, 58, 58, 60], some research has indicated that all truth tellers generally report more details than deceivers, including in Arab and south Asian cultures [25, 26,51, 60]. Given the novelty of this research, rather than hypothesising, we formulated a series of research questions towards advancing pancultural understanding with reference to the real-world challenges, current empirical understanding and associated empirical questions raised by those concerned in developing guidance for practice. It is these questions that guided our paradigm and analysis approach, as follows.

First, we examined lay-persons absolute judgments of truth and lies having seen a single within culture interview conducted in matched first language. Consistent with findings from research with other within cultural groups, we expected a truth bias to emerge, and that confidence would differ across veracity conditions.

Second, we examined subjective assessments of plausibility, believability and detailedness as veracity cues, asking whether variations across deceiver and truthteller accounts are discernible and impactful for guiding veracity judgements having been exposed

to just one interview. Consistent with a very limited amount of previous research, we expected lay-persons to rate liars as less plausible, less believable and less detailed than truthtellers. We use the terms plausible and believable since although virtually synonymous, statements that are believable are *likely* to be correct or true whereas plausible refers to accounts being *reasonable* in terms of being correct or true. It is unclear whether plausible would be robustly and similarly interpreted across cultural groups, since the term has emerged from Western research paradigms. Research from other domains suggests this may not necessarily be the case [61, 62] and so we include believable to cross validate and as a potential additional cue.

Third, we examined the types of cues attended to. We recognise the limited amount of culture specific prior research in this area, nonetheless we expected lay-observers attention would be drawn to behaviours that have been found to be pseudo diagnostic.

Methods and Materials

5. Explain the position taken by the researcher based on earlier studies.

Ethics. This research was ethically approved by the University of Westminster Research Ethics Committee: ETH1617-0528 and the US Dept of Justice FBI institutional Review Board Docket No. 353-16 and was conducted in accordance with the British Psychological Society and Health and Care Practitioner Council codes of ethical conduct. All participants were adults (18 years and older). Consent was gained from each lay person participant via the Qualtrics research platform whereby participants first read an information screen explaining information about this anonymous study and were offered the chance to contact the researchers with any additional questions. Participants were then moved forward to a consent screen. Participants who did not consent (by clicking the ‘I do not consent’ button), were not able to take part in the research and so were directed to a generic ‘thank you and debrief screen’. Those who consented moved through the research process in Qualtrics as described. Consent is stored on the Qualtrics platform. The lay-observer data reported in this

research was collected between 1st June 2023 and 28th August 2023.

Interviewee Participants. For the research reported here, two participant video interviews were selected from a large pool of potential interview data, as described in the Materials section below. These interview data were collected from participants involved in the event described, which was designed to mimic the experiences of some individuals who might later be interviewed as a person of interest.

Culturally matched participants experienced the target event in groups of six. Embedded in each participant group was a culturally matched confederate (C1) playing the role of a participant. Confederate 2 (C2) played the role of researcher (C2). C2 greeted participants and ran the session during which the target event occurred. Participants were primed to be sympathetic to C1 as follows: i) placing C1 within the participant group (C1 arrived and interacted with participants, completed the same tasks, etc.) created conditions for in-group favouritism (Turner, Brown, & Tajfel, 1979); ii) the scenario (the manner in which the event unfolded, and verbal exchanges that took place between C1 and C2 during the event); and iii) C2 acting as researcher in charge of the session, creating a perceived imbalance of social influence (Brewer, 1979), in which C2 was portrayed as more powerful than C1.

Interviewee participants all self-identified as either Arabic, Middle Eastern or West Asian. All were bi-lingual, and born outside of the UK, with Arabic as their first/native language. Participants were paid $30 each to participate. After the stimulus event, prior to being interviewed, participants were instructed to withhold all information about the involvement of C1 and were incentivised to do so by explaining that an additional payment of $60 would be forthcoming dependent upon their interview performance. Not all interviewee participants complied with this instruction, thus the interview data pool comprised interviews with truthtellers and deceivers.

The full research procedure involved four phases for all participants:

1. Participants were provided with an information and consent sheet following which they moved as a group to a seminar room to complete two research questionnaires.
2. While completing the first questionnaire, an unexpected event took place, involving C1 and C2 comprising a verbally aggressive altercation during which a laptop computer was seriously damaged – the entire session, from entering to leaving the event room lasted approximately 25 minutes.
3. Following the event, participants were brought, one by one, into a series of small meeting rooms, where each was informed they would be interviewed individually about the event that had just taken place and given additional verbal instructions by a third confederate (C3) to withhold all information about the presence and involvement of C1 during the interview. Participants were given 20 minutes to formulate a convincing lie script of the event to exclude *all* mention of confederate C1;
4. Participants were individually interviewed. All interviews were digitally audio and video recorded and followed an interview protocol (see Appendix A).

Interviewer. The interviewer was born in the UAE and spoke Arabic as a first language but was fluent in English as a second language. The interviewer underwent a half day of classroom training (given by the first author, an experienced interviewer), which included a detailed explanation of the relevant interview protocol and role-play practice. The interviewer also took part in an additional half-day practice session prior to conducting interviews, which was audio recorded. Detailed verbal feedback was provided on adherence to the protocol. The interviewer was naïve to the design and experimental hypotheses but was provided with the following instructions *'The researcher's computer was seriously damaged during the data collection session. Your job is to interview the people in the room and find out exactly how the damage happened, using the interview protocol'.*

Interview Protocol. Both interviewees were interviewed using the Framed Controlled Cognitive Engagement (Framed - CCE) protocol [3, 37]. Interviews comprised five discrete phases as follows: 1) explain and build rapport, 2) free account, 3) probed questioning, 4) challenge, and 5) closure (for protocol see Appendix A).

Lay Observer Participants. The data collection procedure for lay observers was identical across conditions other than in the cue direction condition where participants were provided with one additional clear instruction prior to viewing the interview, which was to take notice of the amount of detail provided by the interviewee about the event, and to rate the amount of detail on a scale (see materials and Appendix B). Further, that the detail rating should then be used to guide their veracity decision. The research was hosted on Qualtrics. Participants were recruited via Prolific platform where participants were screened for age (+ 18 years) and language (reading and speaking Modern Standard Arabic as first language and English as second). Qualifying participants were then directed to Qualtrics via a one-time link where further demographic questions were included (see Appendix B).

In both conditions (cue direction and no cue direction) all participants were provided with full information about the study, followed by consent information (in Modern Standard Arabic). All participants then completed a series of demographic questions (age, country of birth, parents first language and schooling). Although we used first language (here Arabic) as a proxy for culture, we also asked participants to indicate their cultural identity in terms of their self-identity and self-perception (see Appendix C for self-identity data). Participants were provided with the following written information (in Arabic): '*that the person being interviewed was known to be present during a serious incident where a university laptop was seriously damaged'*. Participants in the cue direction condition were further instructed to '*take note of the degree to which the interviewee includes details such as descriptions of people, places, actions, objects, events, and the timing of events. Think about the degree to*

which the message of the interviewee seemed complete, concrete, striking, or rich in details' [35] It was further explained that they would be asked to rate the interviewee's verbal responses to questions for detailedness after they had seen the video, but before making a veracity decision.

All participants then watched the video (either A or B according to veracity condition, conducted in Arabic). Immediately after having viewed the video interview, participants in the cue direction condition were asked to i) make a detailedness judgement ranging from 1 (no detailed) to 10 (very detailed) and ii) were instructed to use the detailedness judgement score to guide their truth or lie dichotomous decision (where 5 and below should trigger a deception decision). Participants in the no cue direction condition did not receive these instructions and so did not make a detailedness judgment. Rather, they simply made a dichotomous truth or lie decision (see Appendix B for materials).

All participants completed plausibility and believability scales (all ranging from 1 to 10) and were asked to indicate the types of behaviours (verbal only, behavioural only or a mix of both) that had been important in helping them to make a veracity decision. Participants who had indicted verbal behaviours had been important/partially important were asked to think about the way in which the interviewee had verbally responded to questions and to indicate all that applied from a range of known present verbal behaviours. (see Appendix C for materials).

Stimulus Interviews

Each participant listened and watched only one of two information-gathering style interviews. Each interview was conducted in Arabic by the same bilingual interviewer whose first language was Arabic. Both interviews followed the same verbal protocol (see appendix A). In contravention of the confederate (C3) researcher instructions (see procedure), the interviewee in interview A answered all questions truthfully and so did not withhold

information nor formulate a deceptive account of the target event. In interview B (deceiver), as instructed by C3 the interviewee was deceptive by withholding elements of the target event, and so provided an account that was deceptive in part (see procedure). Interview A lasted 11.38 minutes. Interview B lasted 11.35 minutes.

The interviews selected for this research are part of a larger, unpublished dataset and were selected as follows to control for potentially confounding variances. First all interviews in the dataset were translated, transcribed and then coded for target event details. We broadly defined details as verbalised event information provided by the interviewee in response to questions, such as (but not limited to) descriptions of the people involved in the incident in question, clear descriptions of the place and time that the incident took place, information regarding the two confederates actions (including speech), the actions of others present (including speech), the objects involved, and the timing of events. Interview A and B were selected because they differed for event detail verbalised by the interviewee whereby interviewee A provided 41 event details, whereas interviewee B provided 17. Hence, there was an 141% difference in the number of event specific details provided across the two interviews.

Second, both interviewees were well matched. Both were Arabic men, aged 33 and 35 respectively and of a similar west Asian appearance with short dark/black hair. Both were clean shaven but with some dark beard regrowth/shadow, and both were wearing casual clothes (t-shirt & shirt) with no visible tattoos, scars nor other distinguishing markings. Both spoke in Arabic from the start to the end of the interview, were interviewed in the same room and at the same position/angle to the interviewer. Both had been born and raised in Libya and had completed their formal schooling in Libya with English as a second language learned after the age of 16 years. Both interviews were of a similar duration (approx. 11 mins 30 seconds each).

Design

Between subjects 2 Veracity (truth; lie) X 2 Direction (No cue direction; Cue direction) design was employed, collecting dichotomous veracity decision data and scale data for plausibility, confidence and believability from each participant (see procedure and materials).

Lay Observer Participants

A total of 217 bilingual participants, fluent in Modern Standard Arabic as a native/first language, took part as lay observers. One hundred and one females (46.5%), 112 (51.6%) males, and one participant who preferred not to say. Mean age was 27.14 years (SD = 7.12), ranging from 18 to 63 years. While sex and gender are reported, neither are theorized to impact our findings and so sex nor gender-based analyses were not carried out. A-priori power analysis conducted using G*Power 3.1 to determine minimum sample size estimation to detect a practically meaningful medium effect size for applied research of this nature was $N = 179$ (assuming power $= 0.80$, effect size F 0.25, $a = 0.05$). Thus, the obtained sample size of $N = 217$ was adequate given resource constraints and access to Arabic speaking populations and is in line with sample size norms described in many empirical cross-cultural studies similar to the one reported here [23, 26, 55, 56, 64].

Observer participants were only able to take part in one condition and were blind to the existence of the additional studies and conditions and were excluded/screened from other conditions via the Prolific targeted screening and exclusion facility. Two hundred participants were recruited from the general population via Prolific and participated during 1st July and 11th August 2023. The remaining 17 were recruited via social media, word of mouth and snowballing, and participated in the first week in August 2023. Mean age as a function of condition veracity X direction condition did not differ significantly, $F(3, 216) = .149$, $p = .930$, likewise gender distribution, X^2 (3, 216) = 5.049, $p = .536$.

Results

Veracity Decisions

Overall, the dichotomous truth/lie decision data revealed a truth bias. Irrespective of condition (truthteller, deceiver, cue direction, no cue direction), 143 (66%) of observers made a truth decision whereas 74 (34%) made a lie decision. Across conditions, counts of correct and incorrect deception and truth decisions are shown in Fig. 1 (below).

Figure 1. Veracity decision count as a function of condition (N = 217)

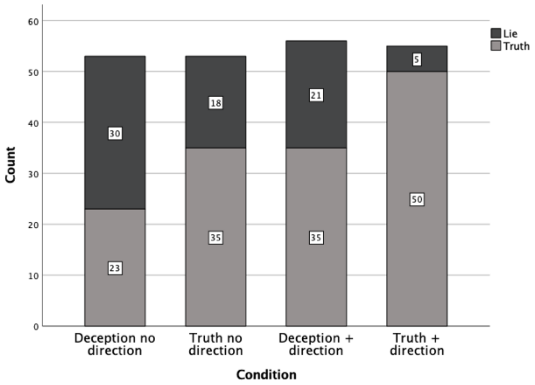

A three-way loglinear analysis of veracity decisions produced a final model that retained all effects. The likelihood ratio of this model was χ^2 (0) = 0, p =1 indicating the higher order model (veracity condition X cue direction condition X veracity decision) was significant, χ^2 (1) = 14.105, p < .001. There was a significant association between veracity condition (truth, deception) and correct veracity decisions, χ^2 (1, 217) = 16.471, p < .001. Eighty-six lay-observers (79%) in the truth condition correctly judged the interviewee was truthful, whereas in the deception condition 51(47%) correctly judged the interviewee as deceptive. The odds ratio of making a correct veracity decision were 19.32 times higher in the truth condition than the deception condition.

Chi-square (χ^2) probability distribution revealed a significant association between cue direction (no cue direction, cue direction) and correct veracity decision, χ^2 (1, 217) = 11.471, $p < .001$. Twenty-six lay-observers (23%) in the cue direction condition correctly judged the interviewee as deceptive whereas in the no cue direction condition 48 (43%) correctly judged the interviewee as deceptive. The odds ratio of correctly deciding the interviewee had been deceptive was 2.56 times higher in the no cue direction condition than the cue direction condition. Loglinear analysis indicated lay observers were more likely to make a correct veracity decision when observing a truthteller than a deceiver and were less likely to correctly judge the interviewee as deceptive when provided with cue directions to make a heuristic detailedness judgement to guide their veracity decisions.

Confidence Scale

There was a significant main effect of veracity condition for confidence ratings (on a scale from 1 to 10, where 1 = at all confident and 10 = completely confident), $F(1, 213) = 7.938$, $p = .005$, $\eta_p^2 = .36$. Participants in the lie condition were less confident ($M = 6.60$, SD = 1.93, 95% CI, 6.27, 6.94) than those in the truth condition ($M = 7.28$, SD = 1.60, 95% CI, 6.95, 7.62). The main effect of cue direction ($M_{\text{no direction}} = 6.76$, SD = 1.89, 95% CI, 6.42, 7.10; $M_{\text{cue direction}} = 7.12$, SD = 1.70, 95% CI, 6.79, 7.45) and the veracity X direction interaction were non-significant, all Fs < 2.243, all *p*s > .136 (see Table 1 below for interaction means, SDs and 95% CIs).

Table 1. Mean interaction confidence scale ratings ($N = 217$)

Condition	Mean (SD) 95% CI
Truth	
Cue Direction	7.34 (1.68) 6.87, 7.81
No Cue Direction	7.23 (1.54) 6.45, 7.71
Deception	
Cue Direction	6.91 (2.07) 6.44, 7.38
No Cue Direction	6.30 (1.74) 5.82, 6.78

Believability Scale

The main effects of veracity and cue direction for believability scale ratings (on a scale from 1 to 10, where 1 = not at all believable and 10 = completely believable) were significant, $F(1, 212) = 18.701$, $p < .001$, $\eta_p^2 = .78$, and, $F(1, 212) = 6.263$, $p = .013$, $\eta_p^2 = .29$, respectively. Participants in the truth condition rated the interviewee as more believable (M = 6.83, SD = 2.31, 95% CI, 6.40, 7.26) than those in the deception condition (M = 5.50, SD = 2.26, 95% CI, 5.07, 5.93) and participants in the cue direction condition rated the interviewee as more believable (M = 6.55, SD = 2.48, 95% CI, 6.13, 6.97) than those in the no direction condition (M = 5.78, SD = 2.21, 95% CI, 5.35, 6.22). The veracity X cue direction interaction was non-significant, F = .062 p = .804 (see Fig 2 below for interaction means).

Plausibility Scale

The main effect of veracity for plausibility scale ratings (on a scale from 1 to 10, where 1 = not at all plausible and 10 = completely plausible) was significant, $F(1, 212) = 15.257$, $p < .001$, $\eta_p^2 = .67$. Participants in the truth condition rated the interviewee as more plausible (M = 6.75, SD = 2.10, 95% CI, 6.34, 7.16) than those in the deception condition (M = 5.59, SD = 2.27, 95% CI, 5.18, 6.01). The main effect of cue direction ($M_{\text{no direction}}$ = 5.93, SD = 2.15, 95% CI, 5.51, 6.35; $M_{\text{direction}}$ = 6.41, SD = 2.35, 95% CI, 6.00, 6.82) and the veracity X cue direction interaction were both non-significant, all Fs < 2.575, all ps > .110 (see Fig 2 below for interaction means).

A repeated measures ANOVA comparing plausibility and believability ratings across conditions revealed non-significant main effects and a non-significant interaction, all Fs < 5.790, all ps, > .017 across conditions.

Figure 2. Believability and plausibility scale interaction means (N = 217).

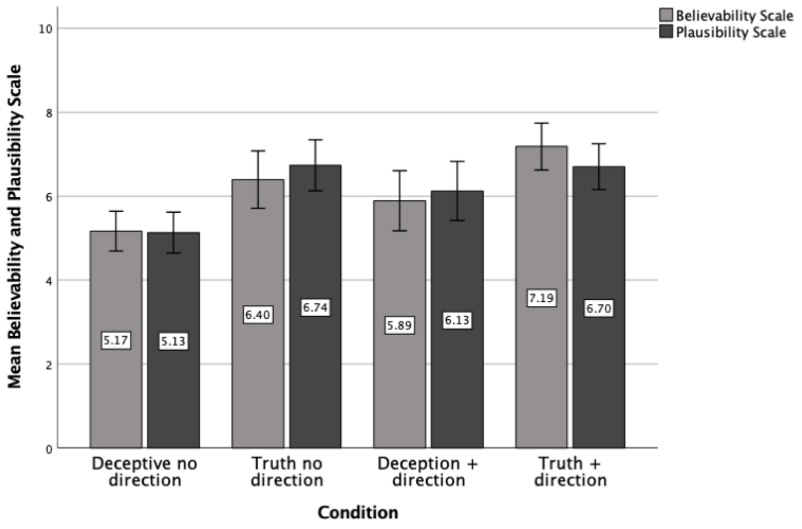

Detailedness

Only participants in the cue direction condition completed a detailedness scale. Analysis as a function of veracity decision revealed participants in the truth condition rated the interviewee's account as significantly more detailed (M = 7.07, SD = 1.46, 95% CI 6.63, 7.46) than those in the deception condition (M = 5.96, SD = 1.78, 95% CI 5.43, 6.39), $F(1, 109) = 14.078$, $p < .001$, $\eta_p^2 = .11$.

Veracity Cue Scale

Analysis of the cues lay observers indicated were important when making a veracity decision revealed significant main effects of veracity and cue direction for cue scale ratings (1 = verbal only, 2 = mainly verbal but some behaviour, 3 = verbal and behaviour equally, 4 = mainly behaviour but some verbal, 5 = only behaviour), $F(1, 213) = 18.775$, $p < .001$, $\eta_p^2 = .81$, and, $F(1, 213) = 17.705$, $p < .001$, $\eta_p^2 = .92$, respectively. Irrespective of cue direction condition, participants in the truth condition (M = 2.79, SD = .97, 95% CI, 2.62, 2.96) indicated that they had *mainly* listened to what the interviewee was saying but took some notice of how the interviewee behaved. Conversely, participants in the lie condition had

mainly taken notice of how the interviewee behaved but took some notice of what the interviewee said (M = 3.32, SD = .91, 95% CI, 3.15, 3.49).

In a similar vein, irrespective of veracity condition, participants in the cue direction condition (who had been cued to note how much detail the interviewee provided and to rate detailedness) indicated they had *mainly* listened to what the interviewee was saying but also took some notice of how the interviewee behaved when making a veracity decision ($M_{\text{cue direction}}$ = 2.77, SD = .82, 95% CI, 2.60, 2.94 – see Table 2 below for interaction means)

Table 2. Cue scale interaction means, SDs and 95% CIs (N = 217).

Condition	Mean (SD) 95% CI
Truth	
Cue Direction	2.50 (.85) 2.26, 2.74
No Cue Direction	3.08 (1.01) 2.83, 3,32
Deception	
Cue Direction	3.04 (.75) 2.79, 3.28
No Cue Direction	3.60 (.99) 3.36, 3.85

Participants in the no cue direction condition *mainly* took notice of how the interviewee behaved but took some notice of what the interviewee said ($M_{\text{no direction}}$ = 3.34, SD = 1.03, 95% CI, 3.16, 3.51). The cue condition X veracity interaction was non-significant F = .001, p = .974.

Verbal Behaviours

Overall, of the 217 lay-observers, 156 (72%) responded that they had primarily or equally attended to verbal behaviours (by responding 1, 2 or 3 on the veracity cue) of which just 10 (4%) responded they had *only* attended to verbal behaviour, 50 (23%) that they had *mainly* listened to what the interviewee said but had taken *some* notice of how the

interviewee behaved, while 96 (44%) stated they had attended equally to what the interviewee said and how they behaved.

Participants indicated which verbal behaviours had been influential when making veracity decision (they were able to indicate more than one behaviour). A Freidman test to rank the verbal behaviours attended to was significant, χ^2 (156) = 21.232, $p < .001$ (see Table 3 below). Overall, self-corrections were ranked as most influential and providing a complicated account was ranked as least influential.

Table 3. Mean ranking, numbers of responses and percentages for verbal behaviours (n = 156)

Verbal Behaviour	Mean Rank	Number (%)
Variously correcting answers	3.94	94 (60.25)
Giving a consistent account	3.55	74 (47.43)
Providing a lot of detail	3.52	72 (46.15)
Giving a simple account	3.44	68 (43.59)
Talking about other people present	3.36	64 (41.02)
Giving a complicated account	3.19	55 (35.25)

Further Freidman tests to rank the verbal behaviours attended as a function of cue direction condition was significant for the no cue direction condition, χ^2 (46) = 20.181, $p <$ .001, (see Table 4) but non-significant for the cue direction group, χ^2 (110) = 9.032, $p = .108$ (see Table 4). Again, self-corrections were ranked as most influential for the non-cue direction group and providing a complicated account was ranked least influential.

Table 4. Mean ranking for verbal behaviours as a function of no cue (n = 46) and cue direction condition (n = 110).

No Cue Direction Mean Rank		Cue Direction Mean Rank	
Variously correcting answers	4.33	Variously correcting answers	3.78
Talking about other people present	3.48	Giving a consistent account	3.64
Providing a lot of detail	3.41	Providing a lot of detail	3.56
Giving a simple account	3.41	Giving a simple account	3.45
Giving a consistent account	3.35	Talking about other people present	3.31
Giving a complicated account	3.02	Giving a complicated account	3.26

Mann-Whitney tests revealed significant differences in verbal behaviours attended too as a function of cue direction condition (direction; no direction) for consistency, U = 1920.00 p = .006 (Mean Rank $_{\text{No direction}}$ = 65.24; Mean Rank $_{\text{Cue direction}}$ = 84.05), complexity, U = 1967, p = .008 (Mean Rank $_{\text{No direction}}$ = 66.26; Mean Rank $_{\text{Cue direction}}$ = 83.62), and detailedness, U = 2044.00, p = .029 (Mean Rank $_{\text{No direction}}$ = 67.93; Mean Rank $_{\text{direction}}$ = 82.94). Participants in the cue direction group attended to all three verbal behaviours more than those in the no cue direction. Mean rankings for all other verbal behaviours were non-significant across the two groups, all Us > 2136.00 ps, > .075, indicating participants attended equally across conditions.

Discussion

Behavioural and verbal norms and expectations vary across cultures and so research understanding truth and lie judgements in non-western cultures plays a central role in reducing misunderstanding and misinterpretation. Towards improved ecological validity we focused on lay-observer (non-student) veracity judgements of participants with Arabic as their native language who variously self-identified as Arabic, Middle Eastern, North African and/or West Asian etc. Furthermore, to improve generalizability we mirrored common real-

world practice whereby our lay-observers had access to just a single interview, following which they were asked to make an absolute veracity judgement.

Consistent with findings from research carried out with North American and Western European participants, and as predicted by Truth Default Theory [66], overall, our data reveals a truth bias whereby 66% of lay participants made a truth judgment. Most interpersonal communication in everyday life is truthful and assuming truthfulness appears pan cultural for within culture non-western native speaker interactions, as reported by others, albeit typically where relative veracity judgments have been made [17, 48, 54, 64,67, 68]. Judgements as a function of veracity condition revealed participants in the truth condition were most accurate whereby 79% correctly judged the interview as truthful. Accuracy was just below chance at just 47% in the deception condition. Again, this pattern of results mirrors the findings of research with North American and Western European observer participants and some non-western native speaker research [43, 69, 70], although little research has investigated truth and lie judgements following a single interaction.

Turning to the impact of instructing observers to take note of verbal detailedness and then to subsequently use these ratings as a rule of thumb to cue a veracity decision. Our results deviate from the findings of [35] where detailedness was an important single heuristic for guiding accurate judgments. Here, relatively our lay observers performed better when judging deception in the absence of a detailedness cue direction (albeit at just below chance). Accuracy dropped considerably to just 23% when instructed to use the detailedness cue. Although our data reveal significant differences in detailedness ratings across the veracity conditions, the mean detailedness rating in the deception condition was 5.96, which is very near to the truth cut-off of 6. Cautious dichotomous decisions in terms of erring on the side of truth as often occurs in situations of uncertainty may account for this result, but this may also be a cultural effect.

Different cultures vary in their linguistic communication style [71, 72]. While cautious dichotomous decisions may have been triggered by the nature of the cut-off instructions, cultural norms may also have played a part in why detailedness may not have been a diagnostic cue here. Participants in the no-cue condition indicted taking note of detailedness even though they were not directed towards any verbal behaviours and so detailedness appears relevant to some degree. However, repetition, re-phrasing and reverse re-phrasing are common persuasive strategies for Arabic speakers, none of which necessarily introduce additional detail across the duration of an interview. This cultural norm may have interfered with participant understanding of the notion of detail and what more or less detail 'looks' like in practice.

Both cue conditions did rank 'corrections' as being the most noted verbal behaviour despite instructions indicting otherwise in the cue direction condition. Corrections may be interpreted as a red flag for Arabic speakers, whereby language is viewed as a 'container' of truth and knowledge and repetitions are common and to be expected. Conversely, alterations and corrections may be uncomfortable and seen as anomalous. It has been suggested that individuals from high-context Asian and Arabic cultures would not naturally expect to be specific when communicating. They may be slower making a point when answering questions than individuals from low context North America and Western Europe cultures. This preference for what is often referred to as roundabout messaging can be even more marked if the topic or 'message' is unpleasant or difficult [25, 80, 81] as was the case here.

The witnessed scenario depicted a verbally aggressive exchange and damage to a university laptop in conditions of perceived imbalance of social influence. Despite instructions to provide as much detail as possible, answering questions may have felt unpleasant and/or difficult and so one might expect verbal accounts to be less detailed *per se*, irrespective of veracity condition. The liar account used for this research was far less detailed

than the truthteller account in terms of the number of event details provided. Directing participants to consider detailedness may have inadvertently alerted observers in the cue condition to note a culturally determined truth cue: less detail being a cultural norm and to be expected particularly when truthfully answering questions about an unpleasant incident.

Where comparative judgements are not possible, our results indicate a lack of detail may be a less valid veracity cue for some non-western cultural groups. Comparative judgements allow observers to naturally notice anomalous or seemingly ‘different’ behaviours across a cohort, which can then be used to guide truth or lie decisions. Differences in levels of detailedness across a within-culture group might be an effective cue in repeated measures contexts, but we did not find this to be a useful heuristic. Others have reported that individuals from some cultures (e.g., Arab and Chinese) naturally provide less detail than individuals from western cultures and so perceptions regarding a lack of detail may not emerge as a pancultural red flag, although more research is needed [5, 26, 28]. Interaction effects highlight observers rated detailedness differently across cue conditions, but these differences did not impact veracity judgments, and detailedness was not ranked as the most important verbal cue behaviour irrespective of cue direction condition.

Ratings of believability and plausibility were used to further understand how observers made their judgements. Although virtually synonymous, given the non-western nature of this research we decided to use both terms for cross validation purposes since plausibility has emerged from North American and Western European research. Main effects for both subjective ratings were consistent with previous research and add to suggestions that plausibility is a cue worth examining [46]. Participants all rated the truthteller as more plausible and more believable than the liar. However, participants in the cue direction condition (who were directed to attend to detailedness) rated both truthteller and liar as more

believable than those in the no cue direction condition, indicating caution when considering subjective measures of believability combined with cueing detailedness.

Previous research has found differences in the believability of individual liars can explain 98% of variance in accuracy of deception detection [54]. It is possible that despite our attempts to match the two interviews, the deceptive interviewee may have had an honest demeanour, albeit this is speculative. Given the attributes of demeanour are complex to specify and were neither considered nor controlled, this finding may be a materials effect. It appears therefore, that plausibility and believability have potential as robust pan cultural subjective assessments, but believability may be more linked to behavioural demeanours which were not the focus of this research.

Our results reveal that participants in the cue condition did as instructed, and had *mainly listened to what the interviewee was* saying but took some notice of how the interviewee behaved when judging veracity. Conversely, participants in the no cue direction condition indicated they *mainly took notice of how the interviewee behaved* but took some notice of what the interviewee said. This latter finding suggests non-verbal indicators may have been effective for correctly identifying truth since overall participants in the no-cue direction condition were more likely to make a correct truth judgment.

Non-verbal gestures are an important aspect of communication in many non-western high context cultures which may explain the importance of this cue for our observers. However, perceptions do not necessarily mirror behaviour, and given the nature of our paradigm there is little reason to question widely reported findings that non-verbal cues are not reliable cues to truth and lies [20, 38, 75, 76] even when participants are trained in identifying them [66, 77]. Most participants took note of non-verbal behaviours to a lesser or greater degree. It is impossible to tease apart the relative contribution of verbal and non-

verbal cues here, thus further research investigating non-western, within-culture non-verbal behaviours is needed to shed more light on this finding.

Supplement this research with formalistic findings, namely the research's keywords that might serve as a guide for problem-solving

Limitations

Better understanding of human behaviours across a broader range of within culture interactions is needed towards diluting western dominated psychological understanding of truth and lies. Furthermore, researchers must consider ecological validity [68]. While our research is novel and relevant to both agendas, as with all experimental research of this nature there are several limitations. Methodological limitations stem from the artificial nature of our judgment task whereby participants were provided with limited detail about the event in question, only viewed one video interview, and their veracity decisions had no ramifications. Future research might consider emphasising the potential implications of accurate versus inaccurate veracity decisions by incentivising participant decision-making perhaps. Allowing participants to view a series of interviews, employing think aloud methods would also help to pick apart the cues noted by this under researched cultural group, particularly the relevance of corrections for example.

Numerous questions emerge centred on the importance or otherwise of corrections, repetitions and detailedness. We did not analyse these linguistic elements in any detail across our larger data set to understand whether the two interviews selected for this research were representative of our Arabic interviews. However, guided by the results of this study, we intend to pursue this aspect of information gathering interviews with Arabic speakers. Finally, we employed an information gathering protocol that includes best practice techniques for gathering IRI and has been used pan culturally in the field [26, 37, 60, 74, 78]. However, for this cultural group alternative interview approaches may prove more effective for leveraging potentially diagnostic veracity signals in more formal contexts.

Conclusion

Behavioural and verbal norms and expectations vary across cultures, however our findings suggest lay observers exhibit similar truth biases and with comparable levels of confidence. Participants in the truth condition were more confident and more accurate, again indicating cues to truthfulness seem more apparent to observers albeit they may not necessarily be quantifiable [38, 79]. Arabic speaking observers exhibited some similarities but also some differences to Western and North American observers in the verbal cues they attended too. Irrespective of the cognitive demands associated with being deceptive linguistic veracity cues maybe culturally specific in terms of attention, despite clear instructions to the contrary and so a one size fits all approach to gathering information may be inappropriate for leveraging culturally salient verbal veracity cues in some instances.

References

1. Innes, M. (2002). The 'process structures' of police homicide investigations. *British journal of criminology*, *42*(4), 669-688.
2. Ratcliffe, J. H., & Guidetti, R. (2008). State police investigative structure and the adoption of intelligence-led policing. *Policing: An International Journal of Police Strategies & Management*, *31*(1), 109-128.
3. Dando, C. J., & Ormerod, T. C. (2020). Noncoercive human intelligence gathering. *Journal of Experimental Psychology: General*, *149*(8), 1435.
4. Loftus, E. F. (2011). Intelligence gathering post-9/11. *American Psychologist, 66*(6), 532.
5. Deeb, H., Vrij, A., Leal, S., Verigin, B. L., & Kleinman, S. M. (2020). When and how are lies told? And the role of culture and intentions in intelligence-gathering interviews. *Legal and Criminological Psychology*, *25*(2), 257-277.
6. Coulthard, M., May, A., & Sousa-Silva, R. (Eds.). (2020). *The Routledge handbook of forensic linguistics*. Routledge.
7. Gudjonsson, G. H. (2012). Investigative interviewing. *Handbook of criminal investigation*, 492-518.
8. Soukara, S., Bull, R., Vrij, A., Turner, M., & Cherryman, J. (2018). What really happens in police interviews of suspects? Tactics and confessions. In *Investigating the Truth* (pp. 206-220). Routledge.
9. Bogaard, G., & Meijer, E. H. (2018). Self-Reported beliefs about verbal cues correlate with deception-detection performance. *Applied Cognitive Psychology*, *32*(1), 129-137.
10. Bogaard, G., Meijer, E. H., Vrij, A., & Nahari, G. (2023). Detecting deception using comparable truth baselines. *Psychology, Crime & Law*, *29*(6), 567-583.
11. Bystranowski, P., Janik, B., & Próchnicki, M. (2024). Uncertainty and Condemnation. An Experimental Study on Lay and Expert Intuitions Regarding the Object of Criminal Punishment. *Law & Social Inquiry*, 1-26.
12. Lindholm, T. (2008). Who can judge the accuracy of eyewitness statements? A comparison of professionals and lay-persons. *Applied Cognitive Psychology: The Official Journal of the Society for Applied Research in Memory and Cognition*, *22*(9), 1301-1314.
13. Walsh, D., Dando, C. J., & Ormerod, T. C. (2018). Triage decision-making by welfare fraud investigators. *Journal of applied research in memory and cognition*, *7*(1), 82-91.
14. Nahari, G. (2012). Elaborations on credibility judgments by professional lie detectors and laypersons: Strategies of judgment and justification. *Psychology, Crime & Law*, *18*(6), 567-577.
15. Elaad, E. (2022). Tunnel vision and confirmation bias among police investigators and laypeople in hypothetical criminal contexts. *SAGE Open*, *12*(2), 21582440221095022.
16. Sternglanz, R. W., Morris, W. L., Morrow, M., & Braverman, J. (2019). A review of meta-analyses about deception detection. *The Palgrave handbook of deceptive communication*, 303-326.
17. Levine, T. R. (2022). Truth-default theory and the psychology of lying and deception detection. *Current Opinion in Psychology*, *47*, 101380.
18. Bogaard, G., Meijer, E. H., Vrij, A., & Merckelbach, H. (2016). Strong, but wrong: Lay people's and police officers' beliefs about verbal and nonverbal cues to deception. *PloSOne*, *11*(6), e0156615.
19. Aamodt, M. G., & Custer, H. (2006). Who Can Best Catch a Liar? A meta-analysis of individual differences in detecting deception. The Forensic Examiner, 15, 7-11.

20. Bond Jr, C. F., & DePaulo, B. M. (2008). Individual differences in judging deception: accuracy and bias. *Psychological bulletin*, *134*(4), 477.
21. Ten Brinke, L., Vohs, K. D., & Carney, D. R. (2016). Can ordinary people detect deception after all?. *Trends in cognitive sciences*, *20*(8), 579-588.
22. Burgoon, J. K., Buller, D. B., Floyd, K., & Grandpre, J. (1996). Deceptive realities: Sender, receiver, and observer perspectives in deceptive conversations. *Communication Research*, *23*(6), 724-748.
23. Castillo, P. A., and Mallard, D. (2012). Preventing cross-cultural bias in deception judgments: the role of expectancies about nonverbal behavior. *J. Cross-Cult. Psychol.* 43, 967–978. doi: 10.1177/0022022111415672
24. Laing, B. L. (2015). *The language and cross-cultural perceptions of deception*. Brigham Young University.
25. Leal, S., Vrij, A., Vernham, Z., Dalton, G., Jupe, L., Harvey, A., & Nahari, G. (2018). Cross-cultural verbal deception. *Legal and Criminological Psychology*, *23*(2), 192-213.
26. Dando, C. J., Taylor, P. J., & Sandham, A. L. (2023). Cross cultural verbal cues to deception: truth and lies in first and second language forensic interview contexts. *Frontiers in Psychology*, *14*, 1152904.
27. Taylor, P., Larner, S., Conchie, S., and Van der Zee, S. (2014). "Cross-cultural deception detection" in *Detecting deception: current challenges and cognitive approaches*. eds. P. A.
28. Taylor, P. J., Larner, S., Conchie, S. M., & Menacere, T. (2017). Culture moderates changes in linguistic self-presentation and detail provision when deceiving others. *Royal Society Open Science*, *4*(6), 170128.
29. Hofstede, G., and Hofstede, G. J. (2005). *Cultures and organizations: software of the mind* (2nd). New York: McGraw-Hill.
30. Alesina, A., & Giuliano, P. (2015). Culture and institutions. *Journal of economic literature*, *53*(4), 898-944.
31. Guiso, L., Sapienza, P., & Zingales, L. (2006). Does culture affect economic outcomes?. *Journal of Economic perspectives*, *20*(2), 23-48.
32. Lytle, A. L., Brett, J. M., Barsness, Z. I., Tinsley, C. H., and Janssens, M. (1995). A paradigm for confirmatory cross-cultural research in organizational behavior. *Res. Organ. Behav.* 17, 167–214.
33. Levine, T. R., Park, H. S., and Kim, R. K. (2007). Some conceptual and theoretical challenges for cross-cultural communication research in the 21st century. *J. Intercult. Commun. Res.* 36, 205–221. doi: 10.1080/17475750701737140
34. Reynolds, S., Valentine, D., and Munter, M. (2011). *Guide to cross-cultural communication*. Upper Saddle River, N.J.: Pearson Education India.
35. Verschuere, B., Lin, CC., Huismann, S. *et al.* The use-the-best heuristic facilitates deception detection. *Nat Hum Behav* **7**, 718–728 (2023). https://doi.org/10.1038/s41562-023-01556-2
36. Vrij, A., Fisher, R. P., and Leal, S. (2022). How researchers can make verbal lie detection more attractive for practitioners. *Psychiatry Psychology Law*, 1–14. doi: 10.1080/13218719.2022.2035842
37. Ormerod, T. C., & Dando, C. J. (2015). Finding a needle in a haystack: Toward a psychologically informed method for aviation security screening. *Journal of Experimental Psychology: General*, *144*(1), 76.
38. Vrij, A., Fisher, R., Mann, S., & Leal, S. (2006). Detecting deception by manipulating cognitive load. *Trends in cognitive sciences*, *10*(4), 141-142.

39. Blandón-Gitlin, I., Fenn, E., Masip, J., & Yoo, A. H. (2014). Cognitive-load approaches to detect deception: Searching for cognitive mechanisms. *Trends in cognitive sciences*, *18*(9), 441-444.
40. Sporer, S. L. (2016). Deception and cognitive load: Expanding our horizon with a working memory model. *Frontiers in Psychology*, *7*, 420.
41. Wielgopolan, A., & Imbir, K. K. (2023). Cognitive Load and Deception Detection Performance. *Cognitive Science*, *47*(7), e13321.
42. Dando, C. J., Bull, R., Ormerod, T. C., & Sandham, A. L. (2015). Helping to sort the liars from the truth-tellers: The gradual revelation of information during investigative interviews. *Legal and Criminological Psychology*, *1*(20), 114-128.
43. Bull, R., van der Burgh, M., & Dando, C. (2019). Verbal cues fostering perceptions of credibility and truth/lie detection. *The Palgrave handbook of deceptive communication*, 691-705.
44. Colwell, K., Hiscock-Anisman, C. K., Memon, A., Taylor, L., & Prewett, J. (2007). Assessment Criteria Indicative of Deception (ACID): An integrated system of investigative interviewing and detecting deception. *Journal of Investigative Psychology and Offender Profiling*, *4*(3), 167-180.
45. DePaulo, B. M., Lindsay, J. J., Malone, B. E., Muhlenbruck, L., Charlton, K., and Cooper, H. (2003). Cues to deception. *Psychol. Bull.* 129, 74–118. doi: 10.1037/0033-2909.129.1.74
46. Vrij, A., Deeb, H., Leal, S., Granhag, P., and Fisher, R. P. (2021). Plausibility: a verbal cue to veracity worth examining? *Eur. J. Psychol. Appl. Legal Context* 13, 47–53. doi: 10.5093/ejpalc2021a4
47. De Los Reyes, A., Lerner, M. D., Keeley, L. M., Weber, R. J., Drabick, D. A., Rabinowitz, J., & Goodman, K. L. (2019). Improving interpretability of subjective assessments about psychological phenomena: A review and cross-cultural meta-analysis. *Review of General Psychology*, *23*(3), 293-319.
48. Akehurst, L., Arnhold, A., Figueiredo, I., Turtle, S., & Leach, A. M. (2018). Investigating deception in second language speakers: Interviewee and assessor perspectives. *Legal and Criminological Psychology*, **23**(2), 230–251.
49. Canter, D., & Alison, L. (Eds.). (2018). *Interviewing and deception*. Routledge.
50. Stromwall, L., Granhag, P. A., & Hartwig, M. (2004). 10 Practitioners' beliefs about deception. *The detection of deception in forensic contexts*, 229.
51. Vrij, A., and Vrij, S. (2020). Complications travel: a cross-cultural comparison of the proportion of complications as a verbal cue to deceit. *J. Invest. Psychol. Offender Profiling* 17, 3–16. doi: 10.1002/jip.1538
52. Levitan, S. I., Levine, M., Hirschberg, J., Cestero, N., An, G., & Rosenberg, A. (2015). Individual differences in deception and deception detection. *Proceedings of COGNITIVE*, 331-348.
53. Levine, T. R., Serota, K. B., Shulman, H., Clare, D. D., Park, H. S., Shaw, A. S., ... & Lee, J. H. (2011). Sender demeanor: Individual differences in sender believability have a powerful impact on deception detection judgments. *Human Communication Research*, *37*(3), 377-403.
54. Levine, T.R., Ali, M.V., Dean, M., Abdulla, A.R., & Garcia-Ruano, K. (2016) Toward a Pan-cultural Typology of Deception Motives, Journal of Intercultural Communication Research, 45:1, 1-12 http://dx.doi.org/10.1080/17475759.2015.1137079
55. Al-Simadi, F. A. (2000). Detection of deceptive behavior: a cross-cultural test. *Soc. Behav. Personal. Int. J.* 28, 455–461. doi: 10.2224/sbp.2000.28.5.455

56. Castillo, P. A. (2015). The detection of deception in cross-cultural contexts. *Understanding facial expressions in communication: Cross-cultural and multidisciplinary perspectives*, 243-263.
57. Hope, L., Anakwah, N., Antfolk, J., Brubacher, S. P., Flowe, H., Gabbert, F., et al. (2022). Urgent issues and prospects: examining the role of culture in the investigative interviewing of victims and witnesses. *Leg. Criminol. Psychol.* 27, 1–31. doi: 10.1111/lcrp.12202
58. Anakwah, N. (2022). 47 Cross-cultural investigative interviews. *The Future of Forensic Psychology: Core Topics and Emerging Trends*.
59. Anakwah, N., Horselenberg, R., Hope, L., Amankwah-Poku, M., & Van Koppen, P. J. (2020). Cross-cultural differences in eyewitness memory reports. *Applied Cognitive Psychology*, *34*(2), 504-515.
60. Dando, C. J., Taylor, P. J., Menacere, T., Ormerod, T. C., Ball, L. J., & Sandham, A. L. (2022). Sorting insiders from co-workers: remote synchronous computer-mediated triage for investigating insider attacks. *Human Factors*, 00187208211068292.
61. Flage, R., Aven, T., Zio, E., & Baraldi, P. (2014). Concerns, challenges, and directions of development for the issue of representing uncertainty in risk assessment. *Risk analysis*, *34*(7), 1196-1207.
62. Glette-Iversen, I., Aven, T., & Flage, R. (2022). The concept of plausibility in a risk analysis context: Review and clarifications of defining ideas and interpretations. *Safety science*, *147*, 105635.
63. Cheng, K. H. W., & Broadhurst, R. (2005). The detection of deception: The effects of first and second language on lie detection ability. *Psychiatry, Psychology and Law*, *12*(1), 107-118.
64. Evans, J. R., Pimentel, P. S., Pena, M. M., & Michael, S. W. (2017). The ability to detect false statements as a function of the type of statement and the language proficiency of the statement provider. *Psychology, Public Policy, and Law*, *23*(3), 290.
65. Primbs, M. A., Pennington, C. R., Lakens, D., Silan, M. A. A., Lieck, D. S., Forscher, P. S., ... & Westwood, S. J. (2023). Are small effects the indispensable foundation for a cumulative psychological science? A reply to Götz et al.(2022). *Perspectives on Psychological Science*, *18*(2), 508-512.
66. Levine, T. R. (2014). Truth-default theory (TDT) a theory of human deception and deception detection. *Journal of Language and Social Psychology*, *33*(4), 378-392.
67. Elliott, E., & Leach, A. M. (2016). You must be lying because I don't understand you: Language proficiency and lie detection. *Journal of Experimental Psychology: Applied*, **22**, 488–499.
68. Wylie, K., Pena, M. M., Miller, K. B., Palmer, M. E., & Tate, E. (2022). Observers' accuracy in detecting deception in non-native speakers versus native speakers: A systematic review. *Journal of Investigative Psychology and Offender Profiling*, *19*(3), 204-220.
69. Masip, J., Garrido, E., & Herrero, C. (2006). Observers' decision moment in deception detection experiments: Its impact on judgment, accuracy, and confidence. *International Journal of Psychology*, *41*(4), 304-319.
70. De Turck, M. A., & Miller, G. R. (1990). Training observers to detect deception: Effects of self-monitoring and rehearsal. *Human Communication Research*, *16*(4), 603-620.
71. Ali, H., & Sayyiyed Al-Rushaidi, S. M. (2017). Translating idiomatic expressions from English into Arabic: Difficulties and strategies. *Arab World English Journal (AWEJ) Volume, 7*.

72. Elhadary, T. (2023). Linguistic and Cultural Differences Between English and Arabic Languages and their Impact on the Translation Process. *International Journal of Language and Translation Research*, *3*(2), 103-117.
73. Cesario, J., Higgins, E. T., & Scholer, A. A. (2008). Regulatory fit and persuasion: Basic principles and remaining questions. *Social and Personality Psychology Compass*, *2*(1), 444-463.
74. Sandham, A. L., Dando, C. J., Bull, R., & Ormerod, T. C. (2022). Improving professional observers' veracity judgements by tactical interviewing. *Journal of Police and Criminal Psychology*, *37*(2), 279-287.
75. Masip, J., & Herrero, C. (2015). Police detection of deception: Beliefs about behavioral cues to deception are strong even though contextual evidence is more useful. *Journal of Communication*, *65*(1), 125-145.
76. Dando, C. J., & Bull, R. (2011). Maximising opportunities to detect verbal deception: Training police officers to interview tactically. *Journal of Investigative Psychology and Offender Profiling*.
77. Hauch, V., Sporer, S. L., Michael, S. W., & Meissner, C. A. (2016). Does training improve the detection of deception? A meta-analysis. *Communication Research*, *43*(3), 283-343.
78. Ewens, S., Vrij, A., Leal, S., Mann, S., Jo, E., Shaboltas, A., et al. (2016). Using the model statement to elicit information and cues to deceit from native speakers, non-native speakers and those talking through an interpreter. *Appl. Cogn. Psychol.* 30, 854–862. doi: 10.1002/acp.3270
79. Nortje, A., & Tredoux, C. (2019). How good are we at detecting deception? A review of current techniques and theories. *South African Journal of Psychology*, *49*(4), 491-504.
80. Korac-Kakabadse, N., Kouzmin, A., Korac-Kakabadse, A., & Savery, L. (2001). Low-and high-context communication patterns: towards mapping cross-cultural encounters. *Cross cultural management: An international journal*, *8*(2), 3-24.
81. Hall, J. A. (1980). Voice tone and persuasion. *Journal of Personality and Social Psychology*, *38*(6), 924.

Appendix A: Interview Protocol

Phase 1 Explain and Rapport

The interviewer explained to the participant that she/he was aware that a laptop had been seriously damaged and so would be asking a series of questions about what had occurred. The interviewer provided a general overview of the interview process and explained four ground rules (report everything; do not guess; say if you do not understand; say if you do not know the answer to a question).

Participants were then offered the chance to ask questions. The interviewer then engaged the interviewee in informal conversation to build rapport. Rapport building conversation continued for a minimum of 2 mins at this point during which the interviewer led the participant to understand the reciprocal nature of the interview, using silences and active listening to encourage the participant to speak/respond following self-disclosure statements made by the interviewer.

Phase 2 Free Account

The second phase commenced with the interviewer providing a positively-framed moral rationale [73]. The following prompts were delivered slowly, with a 5 second pause between each prompt: *"I am going to ask you to tell me in more detail what happened earlier. Telling me about everyone that was involved and everything that happened when the laptop was damaged will* ***make*** *you feel that you are doing something to ensure that innocent people do not end up getting blamed for the damage".*

"I have found that even those people who did not initially want to tell me everything understand that the best way forward is when everyone exercises their responsibility to provide full details".

"Those who have told me everything have done just that – exercised their responsibility, and that is the right and proper thing to do – they have done the right thing"

"Telling me everything is what you ought to do, too. That way I can fully understand what happened. Would you agree"?

"So, if we are in agreement, please have good think about what happened and answer all my questions in as much detail as you can. Thank you"

"What I would like you to do now is to tell me exactly what happened from the time you entered the room until the time you left, in as much detail as possible. Remember the four ground rules I described earlier - report everything; do not guess; say if you do not understand; say if you do not know the answer to a question"

The free account was uninterrupted by the interviewer. Once the interviewee had finished speaking the interviewer silently waited a further 5 seconds (counting silently to 5) before thanking the interviewee and moving into the questioning phase of the interview. During this account the interviewer displayed attentive listening behaviours.

Phase 3 Probed Questions

This phase comprised four information-gathering segments concerning *people, actions, verbal* and *mistakes*.

1. People involved *"Describe to me absolutely everything about everyone involved in the damage to the laptop. Please try and provide as much detail as possible because I was not there, and so I don't know what happened".*
2. Movements. *"Can you talk me through everyone's movements in as much detail as possible. Please try and provide as much detail as possible because I was not there, and so I don't know what happened"*
3. Verbal *"Describe everything that was said by everyone in the room. It doesn't matter if you can't remember everything, but try hard to explain who said what, and who spoke to whom. Thank you"*
4. Mistakes. *'Just before we finish, I wonder, have you left anything out or made any mistakes in what you have told me about what happened in the classroom? Please take the time to think hard about what happened and tell me everything. It is really important that I understand.*

Phase 4 Challenge. Irrespective of information yield and/or interview performance, interviewees were verbally challenged concerning the completeness of the account given thus far, and 'pushed' for more information:

'I think I have a fair understanding of what has happened, but I am not sure that you have told me everything you know. I have interviewed others who were in the room at the same time and they have provided me with more information than you have. It is important that you tell me as much as you can because otherwise I cannot fully understand what has happened. Take a few minutes and have another think about what happened. Tell me everything'.

The interviewer sat silently waiting for the interviewee to respond for up to 10 seconds (counting silently). If the interviewee responded, then the interviewer sat and listened. No extra questions were asked. If the interviewee did not respond after 10 seconds then the interviewer moved seamlessly to the next phase.

Phase 5 Closure This final phase marked the end of the interview. Here the interviewer explained that the interview had now finished and thanked the participant for taking part. The participant was offered the opportunity to ask any questions. The recording device was turned off.

Appendix B: Materials

No-Cue Direction Instructions

Next you will be asked to click on a link, to access a video of an interview, conducted in Arabic.

The interview lasts about 10 mins and concerns a serious incident that took place on a UK University campus. The interviewer is trying to find out what happened and who was responsible.

During a research data collection session, a university laptop computer was seriously damaged.

It is known that the person being interviewed was in the room where the incident occurred and saw what happened.

During the interview the interviewee answers questions and explains what happened. Please take your time to watch and listen carefully because you will then be asked to answer some questions about the interview in two ways.

First, evaluate the answers given by the interviewee when questioned about the serious incident on **detailedness** using a scale from 0 to 10.

1 = (no detailed information was provided) 5 = (some detailed event information was provide) 10 = (a lot of detailed information about the event)

Detailedness is defined as:

“The degree to which the interviewee includes details such as descriptions of people, places, actions, objects, events, and the timing of events; the degree to which the message of the interviewee seemed complete, concrete, striking, or rich in details”.

Link to film

Detailedness judgment scale here (0 to 10)

Now you have made a *detailedness* judgement, decide whether the interviewee is lying or telling the truth using the following rule: If you judged detailedness to be 6 or more, you should determine the interviewee is telling the truth. OR if you judged detailedness it to be 5 or lower, you should determine the interviewee is lying about what happened. Truths are typically more detailed than lies. Based on your detailedness judgement, please indicate whether the interviewee is a truthteller (detailedness of 6 or more) or liar (detailedness of 5 or less).

Liar or Truthteller (forced choice answer)

No Cue Direction Instructions

Next you will be asked to click on a link, to access a video of an interview, conducted in Arabic.

The interview lasts about 10 mins and concerns a serious incident that took place on a UK University campus. The interviewer is trying to find out what happened and who was responsible.

During a research data collection session, a university laptop computer was seriously damaged.

It is known that the person being interviewed was in the room where the incident occurred and saw what happened.

During the interview the interviewee answers questions and explains what happened. Please take your time to watch and listen carefully because you will then be asked to answer some questions about the interview.

Link to film

Liar or Truthteller (forced choice answer)

Appendix C: Observer Cultural/Ethnicity Self Identity Data

**Cultural Self Identity**	**Number (percentage)**
Israeli Arab	27 (12.40)
European Arab	11 (5.11)
African/Black African/North African	63 (29.03)
Arab	42 (19.36)
Middle Eastern	47 (21.66)
Nigerian	12 (5.52)
West Asian	15 (6.92)

Figure
Click here to access/download;Figure;PlosOne Figures.docx

PlosOne Figures

Figure 1. Veracity decision count as a function of condition (N = 217)

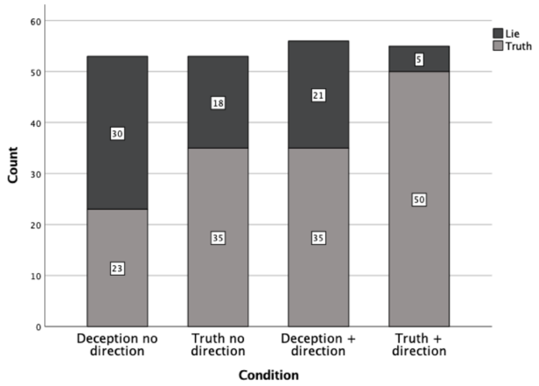

Figure 2. Believability and plausibility scale interaction means (N = 217).

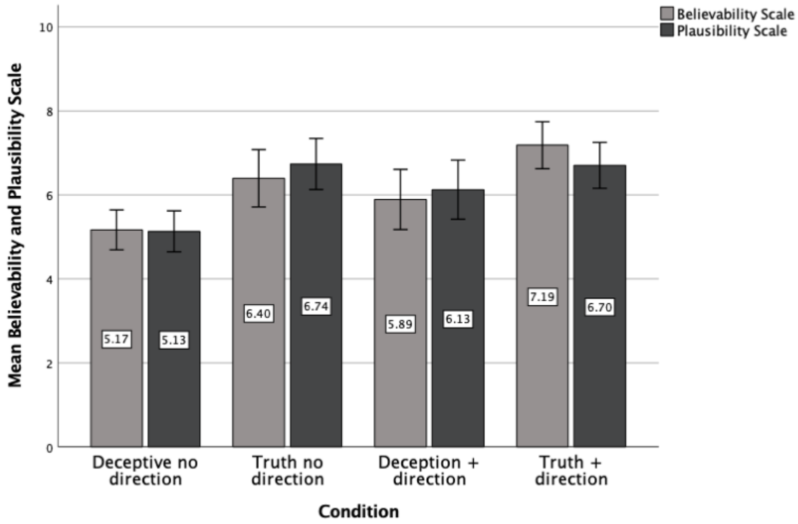

Supplement: Attachment — Submitted filename: PONE-D-24-12342.pdf.Rev.06.07.2024.pdf11.pdf [file pone.0310384.s003.pdf]
