# Supplementary material for: Arabic within culture forensic interviews: Arabic native speaking lay-observer truth and lie accuracy, confidence, and verbal cue selection
Source: PLoS One. 2024 Sep 23;19(9):e0310384. doi: 10.1371/journal.pone.0310384 (PMC11419357; doi:10.1371/journal.pone.0310384)
Supplement: Attachment — Submitted filename: Reviewer Points.docx [file pone.0310384.s004.docx]

**Arabic within culture investigative interviews: Arabic native speaking lay-observer truth and lie accuracy, confidence, and verbal cue selection.**

Dear Editor-in-Chief

Reviewer points as follow:

1. “Introduction” must state the research gap, prior studies on the subject (including those that differ from the prior studies), and the relationship of research problems to the broader interest
2. “The Current Research” must state the focus of research that is different from the previous research
3. “Disussion”: must state the research findings, namely the research's keywords that might serve as a guide for problem-solving, based on literature reviews/theoretical framework.
